# Improving Prediction of Risk of Hospital Admission in Chronic Obstructive Pulmonary Disease: Application of Machine Learning to Telemonitoring Data

**DOI:** 10.2196/jmir.9227

**Published:** 2018-09-21

**Authors:** Peter Orchard, Anna Agakova, Hilary Pinnock, Christopher David Burton, Christophe Sarran, Felix Agakov, Brian McKinstry

**Affiliations:** 1 Pharmatics Edinburgh United Kingdom; 2 Usher Institute of Population Health Sciences and Informatics University of Edinburgh Edinburgh United Kingdom; 3 Academic Unit of Primary Medical Care University of Sheffield Sheffield United Kingdom; 4 Met Office Exeter United Kingdom

**Keywords:** machine learning, telemedicine, chronic obstructive pulmonary disease

## Abstract

**Background:**

Telemonitoring of symptoms and physiological signs has been suggested as a means of early detection of chronic obstructive pulmonary disease (COPD) exacerbations, with a view to instituting timely treatment. However, algorithms to identify exacerbations result in frequent false-positive results and increased workload. Machine learning, when applied to predictive modelling, can determine patterns of risk factors useful for improving prediction quality.

**Objective:**

Our objectives were to (1) establish whether machine learning techniques applied to telemonitoring datasets improve prediction of hospital admissions and decisions to start corticosteroids, and (2) determine whether the addition of weather data further improves such predictions.

**Methods:**

We used daily symptoms, physiological measures, and medication data, with baseline demography, COPD severity, quality of life, and hospital admissions from a pilot and large randomized controlled trial of telemonitoring in COPD. We linked weather data from the United Kingdom meteorological service. We used feature selection and extraction techniques for time series to construct up to 153 predictive patterns (features) from symptom, medication, and physiological measurements. We used the resulting variables to construct predictive models fitted to training sets of patients and compared them with common symptom-counting algorithms.

**Results:**

We had a mean 363 days of telemonitoring data from 135 patients. The two most practical traditional score-counting algorithms, restricted to cases with complete data, resulted in area under the receiver operating characteristic curve (AUC) estimates of 0.60 (95% CI 0.51-0.69) and 0.58 (95% CI 0.50-0.67) for predicting admissions based on a single day’s readings. However, in a real-world scenario allowing for missing data, with greater numbers of patient daily data and hospitalizations (N=57,150, N^+^=55, respectively), the performance of all the traditional algorithms fell, including those based on 2 days’ data. One of the most frequently used algorithms performed no better than chance. All considered machine learning models demonstrated significant improvements; the best machine learning algorithm based on 57,150 episodes resulted in an aggregated AUC of 0.74 (95% CI 0.67-0.80). Adding weather data measurements did not improve the predictive performance of the best model (AUC 0.74, 95% CI 0.69-0.79). To achieve an 80% true-positive rate (sensitivity), the traditional algorithms were associated with an 80% false-positive rate: our algorithm halved this rate to approximately 40% (specificity approximately 60%). The machine learning algorithm was moderately superior to the best symptom-counting algorithm (AUC 0.77, 95% CI 0.74-0.79 vs AUC 0.66, 95% CI 0.63-0.68) at predicting the need for corticosteroids.

**Conclusions:**

Early detection and management of COPD remains an important goal given its huge personal and economic costs. Machine learning approaches, which can be tailored to an individual’s baseline profile and can learn from experience of the individual patient, are superior to existing predictive algorithms and show promise in achieving this goal.

**Trial Registration:**

International Standard Randomized Controlled Trial Number ISRCTN96634935; http://www.isrctn.com/ISRCTN96634935 (Archived by WebCite at http://www.webcitation.org/722YkuhAz)

## Introduction

### Background

Exacerbations of chronic obstructive pulmonary disease (COPD) are a major cause of acute hospitalizations. Prompt intervention with antibiotics and corticosteroids may prevent admissions and improve quality of life [1,2], but difficulties in recognizing early symptoms of deterioration [3] often result in delays in accessing care [2,4] and starting treatment. Telemonitoring of symptoms and physiological measurements has been advocated to facilitate early identification and treatment of exacerbations. However, despite patients’ perceptions [4], the evidence from randomized controlled trials that telehealth prevents admissions is less than convincing [5-9]. One reason for this is that, far from clarifying the early detection of exacerbations, previously employed algorithms (typically based on international definitions of exacerbations [10]) generate frequent, clinically unnecessary alerts [11].

New symptom-based algorithms have been designed to improve identification and assessment of established exacerbations [12,13]. There is some evidence that a composite measure combining oxygen saturation and heart rate with symptoms may predict deteriorations requiring treatment with antibiotics or corticosteroids [14], although these physiological measures have marked day-to-day variation, which may obscure subtle changes due to early exacerbations in individual patients [15]. The optimal algorithm is thus not yet clear.

Recently, there have been major advances in developing computational and statistical methods for analyzing noisy, incomplete data, broadly described as machine learning and data mining [16,17]. When applied to predictive modelling, such methods can determine patterns of risk factors useful for improving the quality of predictions. This is in contrast to conventional algorithms, which typically use a small number of established risk factors. However, these techniques are not yet in use for predicting hospital admissions for COPD in patients undergoing telemonitoring.

### Objective

Data from the Telescot COPD telemonitoring program [4,11] included daily symptom and physiological measures, which could be linked with health care use (consultations, prescription of medication, and hospital admission); baseline data on age, severity of COPD, comorbidity, and anxiety and depression scores; and contextual data (such as weather conditions from the Met Office (the United Kingdom [UK] meteorological service) [18]. Using machine learning and high-dimensional data mining, we aimed to use this large dataset to identify patterns predictive of hospital admissions or decisions to start corticosteroids.

## Methods

The Telescot COPD trial (ISRCTN 96634935) [11] was undertaken in 2009-2011 preceded by a pilot study [4] in 2008 in Lothian, Scotland. Ethical approval was granted by the Lothian research ethics committee (reference 08/S1101/60), with UK National Health Service (NHS) management approval from NHS Lothian, Scotland.

### Datasets and Handling

The telemonitoring database of day-to-day symptoms and physiological measures from the Telescot COPD trial [11] and pilot study [4] was held by the NHS. These were linked in the Lothian safe haven with trial data held by the research team and hospital admission data. Datasets were deidentified before analysis.

#### The Telemonitoring Dataset

The Telescot COPD program [4,11] included 146 patients who had moderate to severe COPD (forced expiratory volume in the first second of expiration [FEV_1_] and forced vital capacity both <70%) and at least one admission to hospital in the previous year for an exacerbation of COPD. They recorded data with some gaps over an average of 16 months. Patients were asked to provide daily symptoms and physiological readings (pulse and oxygen saturation, and a subset of the study population also provided spirometry data on a less regular basis) and to record antibiotic and corticosteroid use. The symptom score was based on the presence of major symptoms (scored 2) or minor symptoms (scored 1) based on the work of Anthonisen et al [19] and others [2,10,11,15] (see Textbox 1). Data were checked daily by a respiratory professional or trained telehealth monitor, and patients were contacted if their symptom score rose above 5. Acceptable ranges for pulse rate and oxygen saturation were set individually, and patients were contacted if readings fell beyond these ranges.

**Definitions of chronic obstructive pulmonary disease exacerbation onsets on day *t* used as predictors of hospital admissions on day *t*+1. Note that the last 3 definitions cannot be used for this evaluation unless an early detection can be made, as they detect an onset of an exacerbation with a 1-day delay. For these definitions, we report an approximate upper bound on the predictive performance under the assumption that the exacerbations can be detected.**

1. **Major symptoms**
  - Breathlessness, sputum color, and sputum amount.
2. **Minor symptoms**
  - Cold, wheeze, sore throat, cough, and fever.
3. **Symptom counts**
  - nMajor = number of major symptoms present on day *t*,
  - nMinor = number of minor symptoms present on day *t*,
  - nAll = nMajor + nMinor.
4. **Definitions**
  - Definition 1 (after Anthonisen et al. [19]): nMajor≥2.
  - Definition 2 (modification of Rodriguez-Roisin [10]): nAll≥5.
  - Definition 3 (modification of Exacerbation 1 as in Seemungal et al. 2]): define a 'bad day' as one where (nMajor≥2) or ([nMajor=1] and [nMinor≥1]). An exacerbation is said to occur on day *t* if days *t* and *t* +1 are bad, but days *t* –1 and *t* –2 are not bad.
  - Definition 4 (modification of Seemungal et al. 2] as in Burton et al. 15): Like Definition 3, but a bad day is defined as one where (nMajor≥1) and (nAll≥3).
  - Definition 5 (after Pinnock et al. 11): An exacerbation is said to occur on day *t* if:
    - (nAll≥5) on day *t*, or
    - (nAll=4) on day *t* and (nAll≥4) on day *t* +1.

#### Trial Data

Baseline trial data on demographic characteristics, body mass index, spirometry, Medical Research Council Dyspnoea Scale [20], Hospital Anxiety and Depression Scale [21], St George’s Respiratory Questionnaire [22], previous hospital admissions, and comorbidity were also available. At the end of the trial, we searched hospital records for admissions during the trial, and 2 clinicians determined whether the admission was due to COPD, partly due to COPD, or unrelated to COPD.

#### Met Office Health Forecasting Data

The UK Met Office Healthy Outlook service uses a rule-based model, combining observed and forecast parameters, including season, humidity, temperature, air quality, and rates of influenza-like illness to issue forecasts. These have been shown to provide a 10-day warning of periods of higher risk of COPD exacerbations at a population level [18], but it is unclear whether this is predictive at an individual level. We hypothesized that including Met Office data along with telemonitoring and baseline data would improve the algorithm’s prediction. We therefore combined the telemonitoring dataset with the Met Office COPD health forecasting dataset. This consisted of the outputs of the Met Office’s Healthy Outlook COPD alert algorithm [18], maximum and mean temperatures in the last 48 hours, and 3 binary temperature indicators (mean temperature <2°C, maximum temperature <4°C, and maximum temperature <7°C).

#### Choice of Outcomes

We gave patients taking part in the study an individualized action plan, which typically advised starting antibiotics if their symptom score exceeded 5, so antibiotic courses were very frequent events. As a proxy for more serious exacerbations, we tested the prediction of two main outcomes: admission to hospital for COPD and initiation of oral corticosteroid treatment.

#### Preprocessing

We defined *patient episodes* as sliding windows of patient-generated data for a fixed number of consecutive days up to the current day (inputs), linked to the admission or corticosteroid outcome on the following day (output).

We considered the simple score-counting algorithm in the complete-data setting, where we used only episodes without any missing symptom variables to compute risk scores for 1- or 2-day windows. Additionally, we evaluated the score-counting algorithms and the machine learning models in the imputation setting using identical patient episodes, where we imputed each missing variable by copying the last observation of that variable for that patient for up to 15 days. We excluded episodes where the outcome variable was missing and patient episodes where we could not impute the observations due to long windows of no provided data. Where we used imputation, for each variable in the patient episode, we defined an auxiliary indicator to encode whether the input variable was imputed or provided by patient; we used these auxiliary variables as additional inputs into the machine learning models. Note that the sample size and the number of admissions for the imputation setting were higher than those for the complete-data setting. For example, if some measurements were not reported prior to a hospital admission, then we excluded the episode from the complete-data analysis, but we could retain it in the imputed setting when the reported variables were exact and the missing variables were imputed.

### Data Analysis

We identified a large number of potentially predictive features by using established data mining techniques (see below) and tested them in combinations using nested cross-validation procedures, where we selected and extracted the feature by using only the inner training folds of data. Because data were incomplete, we conducted separate analyses (1) limited to time periods with no missing data, and (2) from all time periods with imputation of missing data.

#### Identification of Novel Features

For each patient, we constructed up to 153 predictive patterns (features) from symptom, medication, and physiological measurements, by using feature extraction techniques for time series [23,24], hypothesized to be predictive of the future events [2] (see Multimedia Appendix 1). The exact number varied between the complete and imputed settings and depended on which types of variables (telemonitoring, weather, and their combinations) we used as inputs. We imputed variables measured at baseline by using population medians for the continuous variables or population modes for the categorical variables, and we assumed the variables to be fixed (stationary) throughout the study. We used the resulting variables to construct predictive models fitted to the training sets of patients. We used only the past, and not the future, variables for imputing the missing variables or constructing the time-series features for each patient episode. The resulting variables were combined to learn additional features in the hidden layers (neural nets), used for computing feature-space similarity functions (nonparametric methods), or combined with feature selection by filtering [25] to set priors on hyperparameters (adaptive regularized classifiers) during training. When we used the output variables directly or indirectly to select or extract the features during training, we ensured that the procedure was nested within the training folds, so that the data used for the evaluations remained unseen.

#### Standard Exacerbation Models

We considered several definitions of exacerbations based on the criteria of Anthonisen et al [19] and clinical guidelines [26] and used in studies on COPD exacerbations [2,10,11,15]. Major symptoms were changes in patients’ self-reported *breathlessness*, *sputum color*, and *sputum amount*, and minor symptoms were cold, wheeze, sore throat, cough, and fever. Using definitions from the literature, we considered 5 definitions of exacerbation (Textbox 1). We evaluated the onsets of exacerbations on a given day (*t*) as predictors of admissions the following day (*t* +1). Note that, from the considered definitions, only definitions 1 and 2 could be used for this type of evaluation. For example, definition 3 is defined as the presence of at least two consecutive days of major symptoms, or one major and at least one minor symptom, with the exacerbation onset taken to be the first day when the symptom criteria are met [1,2,27], whereas definition 4 is its slight modification [15]. Thus, for definitions 3 to 5, by using the exacerbation indicator on day *t* as a marker of an admission on day *t* +1, we evaluated an upper bound on the predictive performance under the assumption that these exacerbations can be detected early (eg, by making accurate predictions of the future symptoms).

#### Novel Predictive Modeling

We assessed how well we could predict hospital admissions and decisions to start corticosteroid treatment in patients undergoing telemonitoring using the extracted features. We considered several types of models. (1) Nonparametric predictive methods, such as sparse maximum-margin classifiers [16,28,29]: these approaches allow for complex mappings from covariates to target outcomes to obtain high-quality “black-box” predictions. (2) Regularized classifiers based on the adaptive extensions of elastic nets [30]: in low dimensions, these methods have the advantage of generating intelligible predictions, but they may sometimes result in lower predictive performance than nonparametric methods or ensembles due to rigid constraints on the mappings between covariates and outcomes. (3) Ensembles of boosted classifiers [31] that we expected to be well suited for dealing with highly imbalanced datasets such as ours (where the number of episodes corresponding to COPD admissions was several orders of magnitude lower than the number of episodes without admissions). (4) Long short-term memory multitask neural network models: these methods are state-of-the-art for speech recognition, where very large datasets are available [32]. However, we found their performance to be only a little better than that of the other models for our smaller incomplete imbalanced dataset. We considered these models using the preprocessing strategy discussed above and using training by a variant of back-propagation for recurrent networks.

We repeated the procedure by considering features occurring (1) 24 hours prior to hospitalization or earlier, and (2) 24 hours prior to the decision to start corticosteroids or earlier. We fitted models 1 and 2 by regressing the outcomes on telemonitoring only (physiological, medication, and symptom variables), weather variables only, and telemonitoring and weather variables jointly. We used the more computationally expensive models (3 and 4) for regressing the outcomes on the telemonitoring variables in the imputed scenario. Hyperparameters were learned by the grid search (models 1 and 2) or by random search (models 3 and 4) over inner folds in the nested cross-validation procedure.

We compared these methods with the conventional algorithms using multiple definitions of exacerbations from Textbox 1 as predictors of the future clinical admissions and corticosteroid therapy.

#### Validation of Novel Predictive Models

To test this range of models, we used *k*-fold cross-validation, in which we split the data into *k* disjoint subsets (“folds”) of equal size, and fitted the models repeatedly to *k* –1 training folds, evaluating them on the remaining test fold. The procedure was repeated *k* times, and the overall performance was evaluated by aggregating the results across the test folds. During the nested cross-validation, we performed the cross-validation procedure for each choice of test data in a nested loop, where we used the inner training folds for feature extraction and selection and for estimating model parameters, we used the inner validation folds for estimating hyperparameters (such as the degree of model complexity), and we used the outer test folds purely for the performance evaluation. In our implementation of the procedure, we ensured that the test outer folds were made up of individuals who did not appear in the training sets or the inner folds (ie, we used no patient episodes for individuals from test datasets as any part of the training data). Thus, we used the outer test sets of patients purely for evaluations, and not for variable selection, parameter learning, or hyperparameter learning. We evaluated the predictive performance expressed as the aggregated area under the receiver operating characteristic curve (AUC), a calibration-invariant measure of predictive performance of binary classifiers. The aggregation was achieved by merging the predictions of the classifiers across the test folds and by averaging the merged AUC across multiple repetitions of cross-validation with the random fold partitions.

#### Experimental Comparison

We excluded 11 individuals with more than 95% missing data and analyzed data for 135 individuals who provided symptoms and physiological measurements regularly. We chose the outer folds to have approximately the same number of patient episodes, although an equal splitting could not be guaranteed, as patients had unequal numbers of the reported measurements. We used 10 inner and 10 outer folds of the nested cross-validation procedure for all but the most computationally expensive models. To evaluate the variation in the performance, we used 10 runs of the nested cross-validation with different training or test fold partitions.

We evaluated simple score-counting algorithms that did not need long series of past symptoms to generate predictions, both in the complete and in the imputation scenarios. We used machine learning models that needed longer sequences of partially missing past observations in the imputation scenario. In that scenario, we excluded all patient episodes that we could not impute according to the considered procedure due to too much data being missing. For a fair comparison of multiple models, we ensured the consistency of the imputations and patient episodes across the folds.

## Results

### Predicting Hospital Admissions of Individuals

In the complete-data scenario, we evaluated how well the traditional definitions of exacerbation onset on one day predicted 24-hour hospital admissions the following day, using the definitions from Textbox 1. Depending on the choice of the algorithm, we had between 14,106 and 17,610 patient episodes, and between 8 and 17 hospital admissions. We obtained the best predictions by using definition 5 (mean AUC 0.657, 95% CI 0.523-0.792, N=16,170 patient episodes, where we computed the error bars on the AUC as the consensus estimate of the methods of empirical resampling, Chebyshev, and DeLong and colleagues [33]; Table 1); however, we based this estimate on a dataset with only N^+^=9 admissions. Additionally, using this definition, an exacerbation starting on one day could only be detected when the score remained elevated the following day (see Textbox 1), making it impractical for predicting an admission on the second day. Score-counting algorithms definitions 1 and 2, where onsets of exacerbations are computed on a single day, resulted in the AUC estimates of 0.600 (95% CI 0.509-0.692) and 0.578 (95% CI 0.496-0.672), respectively, for N=17,610 episodes and N^+^=17 admissions (Table 1).

When evaluated in the pragmatic imputed-data scenario allowing for missing data, with a greater number of patient episodes (N=57,150) and a greater number of hospital admissions preceded by the symptom and physiological measurements (N^+^=55), the performance of all the traditional definitions of exacerbation dropped to near random. For example, for definition 2, we obtained an AUC of 0.524 (95% CI 0.486-0.544); see Table 1. The most likely reason for this drop was the need to rely on a simple imputation strategy due to the limited availability of daily symptom data on the days preceding hospital admissions.

Machine learning models demonstrated significant improvements in the prediction of future admissions over the traditional symptom-counting methods. Working with the imputed-data scenario, the best machine learning model (neural net) using telemonitoring data resulted in the aggregated AUC of 0.740 (95% CI 0.673-0.803) evaluated on test data for N=57,150 episodes, N^+^=55 admissions (Table 1). The other machine learning models had similar performance, with the mean aggregated AUC of 0.721-0.738, which shows that the improvement over symptom scores could be achieved across a range of models (see Multimedia Appendix 2). To achieve an 80% true-positive rate (sensitivity), the traditional algorithms were associated with an 80% false-positive rate (20% specificity); our algorithm halved this rate to approximately 40% (specificity around 60%).

Adding the weather data (the Healthy Outlook criterion and the additional weather-related variables) to the telemonitoring measurements resulted in no significant improvement in the predictive performance of the best model, with the aggregated AUC of 0.739 (95% CI 0.685-0.794, N=57,150, N^+^=55). This cannot be explained by the weather variables being correlated with the telehealth variables, as the best model using the weather data only had the near-random AUC of 0.526 (95% CI 0.504-0.548, N=107,078, N^+^=151).

The best model for admissions refitted to the entire dataset following the model selection used 135 variables and was difficult to characterize. By linearizing its outputs, we found that the factors contributing most to the predictions included all 3 groups of variables collected by telemonitoring, together with current smoking status: current symptoms, current and delayed physiological measures, and current and delayed self-reported medications.

**Table 1:** Predictive accuracy of hospital admission and use of corticosteroids of different definitions of exacerbation.

Description		Practical	AUC^a^ (empirical 95% CI)	Events, N^+^	Samples, N
**Prediction of 24-hour admissions using exacerbation definitions, *complete* data**					
	Definition 1	Yes	0.600 (0.509-0.692)	17	17,610
	Definition 2	Yes	0.578 (0.496-0.672)	17	17,610
	Definition 3	No	0.553 (0.440-0.666)	8	14,106
	Definition 4	No	0.490 (0.424-0.556)	8	14,106
	Definition 5	No	0.657 (0.523-0.792)	9	16,170
**Prediction of 24-hour admissions using exacerbation definitions, *imputed* data**					
	Definition 1	Yes	0.513 (0.477-0.551)	55	57,150
	Definition 2	Yes	0.524 (0.486-0.544)	55	57,150
	Definition 3	No	0.496 (0.471-0.521)	55	56,702
	Definition 4	No	0.505 (0.473-0.536)	55	56,702
	Definition 5	No	0.517 (0.479-0.555)	55	57,150
**Prediction of 24-hour corticosteroid decisions using exacerbation definitions, *complete* data**					
	Definition 1	Yes	0.655 (0.630-0.679)	238	9768
	Definition 2	Yes	0.605 (0.581-0.628)	238	9768
	Definition 3	No	0.568 (0.544-0.592)	178	8489
	Definition 4	No	0.544 (0.522-0.567)	178	8489
	Definition 5	No	0.646 (0.622-0.670)	237	9322
**Prediction of 24-hour corticosteroid decisions using exacerbation definitions, *imputed* data**					
	Definition 1	Yes	0.660 (0.639-0.681)	316	13,899
	Definition 2	Yes	0.605 (0.585-0.625)	316	13,899
	Definition 3	No	0.564 (0.543-0.586)	228	10,442
	Definition 4	No	0.543 (0.524-0.564)	228	10,442
	Definition 5	No	0.647 (0.626-0.668)	316	12,477
**Prediction of 24-hour admissions using machine learning models, *imputed* data**					
	Machine learning model	Yes	0.740 (0.673-0.803)	55	57,150
**Prediction of 24-hour corticosteroid decisions using exacerbation definitions, *imputed* data**					
	Machine learning model	Yes	0.765 (0.738-0.791)	316	13,503

^a^AUC: area under the receiver operating characteristic curve.

### Predicting Peaks in Symptom Scores in Populations

The Healthy Outlook [18] algorithm and the weather variables did not improve the quality of predictions of hospital admissions for individuals in our dataset. However, at the population level we found that, over some contiguous time periods, predominantly during fall and winter, prediction of the 2-week population-averaged baseline-adjusted symptom score using the Healthy Outlook variables outperformed the prediction of the simple delayed baseline-adjusted symptom score. The Spearman correlation between the true and the predicted outcomes over the test data folds increased from 0.44-0.55 (the lagged heuristic) to 0.66-0.75 (Healthy Outlook), and the Kendall rank correlation increased from 0.27-0.38 to 0.44-0.52. See Multimedia Appendix 1 for additional detail.

### Predicting Individuals Starting Corticosteroids

In contrast to the prediction of hospital admissions, the standard score-counting algorithms were moderately predictive of decisions to start corticosteroid treatments, both in the complete-data and in the imputed-data scenario. Here, we included in the analysis only episodes where patients reported not taking corticosteroids on the first day of the exacerbation. The onset events were defined as taking corticosteroids on the following day. Using definition 1 (Textbox 1), we obtained an AUC of 0.655 (95% CI 0.630-0.679) for the complete-data scenario with N=9768 episodes and N^+^=238 corticosteroid therapy onsets (Table 1). In the imputed-data scenario, we obtained an AUC of 0.660 (95% CI 0.639-0.681) with N=13,899 episodes and N^+^=316 corticosteroid therapy onsets. Although the machine learning models helped to improve the predictions, leading to an AUC of 0.765 (95% CI 0.738-0.791) on the test datasets, this improvement was relatively lower than in the case of predicting the admissions. The algorithm for predicting corticosteroid onsets (a nonparametric model) used 153 features, where the most important one, as suggested by linearizing, was the total symptom score on the current day.

## Discussion

### Principal Results

In the context of telemonitoring, traditional algorithms of predicting exacerbations with imputation of missing symptom data were no better than chance when they were used for predicting a COPD admission over the subsequent 24 hours, and were only a little better than chance in the subset with complete data provided by patients. The performance of machine learning algorithms was considerably more accurate and, in practice and subject to some conditions, would have halved the number of false alerts in comparison with the traditional method (see Multimedia Appendix 1 for additional detail). The algorithm readily identified those at high and low risk of admission, suggesting that, in a resource-constrained environment, a simple triage strategy for targeting additional care could be based on using the output of our method. Adding meteorological data did not significantly enhance the accuracy of the model at an individual level, although it did so, to some extent, at a group level for the prediction of average baseline-adjusted symptom scores, which could be of value to service planners. We found that both the standard symptom-counting algorithms and the machine learning algorithms were reasonably accurate for predicting the decision to start corticosteroids within 24 hours.

### Limitations

Despite the Telescot COPD trial [11] being one of the largest individually randomized trials of telehealth in COPD, the absolute number of admissions immediately preceded by a complete record of physiological and symptom variables was relatively small, which may have reduced the reliability of the algorithm.

The lack of a gold standard definition for what constitutes an exacerbation is a challenge to research in this area. Many mild to moderate exacerbations were defined by medication use, and patients’ individualized management plans advised commencement of antibiotics with an increase in symptoms (eg, if their sputum was dark green). Some also kept corticosteroids, which they took if they were very breathless or wheezy. This self-management may have interfered with what would otherwise have been the natural history of the exacerbation, reducing the relationship between some symptoms and signs and the outcome (hospital admission), but potentially strengthening the relationship between some components of the algorithm and decision to start corticosteroids. Nonetheless, we find the fact that the machine learning algorithm can predict future admissions despite adjusting for self-reported medications to be encouraging.

One methodological limitation of our approach is its reliance on cross-validation, rather than multiple independent cohorts, for evaluations of the predictive performance. In addition to ignoring possible covariate or distribution shifts across multiple cohorts, another well-known disadvantage of cross-validation is the complexity of approximating confidence intervals of the performance measures [34], especially for small or imbalanced datasets. The use of a resampling approach such as cross-validation was unavoidable given the small number of large telemonitoring trials for COPD. Further validations in unrelated datasets will be needed to confirm our findings. One strength of our approach is the use of complementary machine learning methods in the derivation of the optimal algorithm and consistency of the findings across the methods. The considered methods included regularized parametric and kernel methods, boosting, and representation learning. A limitation of our approach is its reliance on fixed-length feature vectors extracted from time-series data, rather than variable-length predictors. We argue that, although there have been some recent works on using variable-length approaches for time-series predictions [35], they demonstrated superior performance over other methods when the number of cases exceeded ours by several orders of magnitude, and they were not extensively compared with sparse classifiers reliant on imputation methods. The closest match to such models from those we considered—the long short-term memory with the imputation strategy described above—did not improve on the other models. Handling the systematic missingness in variable-length conditional models is an actively researched area that will be considered in the future, and which is likely to become useful once bigger telemonitoring datasets are collected. In this study, we used imputation by forward-feeding, which is arguably one of the most practical approaches at the point of inference when access to past data is limited; other techniques may potentially be considered.

The aim of this study was to demonstrate the potential of machine learning for predicting COPD admissions and corticosteroid use, not to elucidate the effects of each feature or combination of features under different adjustments. Modern artificial intelligence methods for predicting clinical events use hundreds or even thousands of features to predict clinical outcomes [34,36]. Due to complex architectures and interactions between multiple variables, it is challenging to estimate the effects of each feature [37,38]. In this study, we investigated the effects only of classes of variables (telehealth, weather-related, and their combinations) rather than each single variable. This is a general limitation of high-dimensional methods; future work is needed to investigate the marginal and conditional effects, and a validation in a device trial will be needed prior to translation to clinical practice.

A limitation of our work is that some of the measures were available at only 1 or 2 time points (eg, anxiety and depression scores, quality of life, exercise or physical activity data, and smoking status were assessed at the beginning and end of the 1-year trial), and time-series data might have been more informative. Other multicomponent scores known to be predictive of COPD outcomes (such as the body mass index, obstruction, dyspnea, exercise index [39] or dyspnea, obstruction, smoking, exacerbation index [40]) might have been useful predictors, as would serial FEV_1_ and more detailed serial information on medication changes. Our machine learning platform is extendable to such new types of data sources that may include systematic or informative missingness, which is the strength of the approach.

### Comparison With Prior Work

Interest in the development of more accurate predictive algorithms using machine learning is increasing; Sanchez-Morillo and colleagues [41] in a recent review concluded that, while some of these show promise, they have been based on relatively small numbers of patients and events [42,43]. They require validation in larger samples of patients, for longer periods of time. The closest to ours is probably the very recent work of Shah et al [44], who used logistic regression to predict future exacerbations and showed that using pulse rate, oxygen saturation, and respiratory rate (from a pulse oximeter) showed improved predictivity when compared with traditional algorithms of COPD exacerbations. Our result in respect of the value of meteorological data is consistent with the work of Steventon et al [45] on the impact of Healthy Outlook on admission rates.

### Conclusions

The early detection and management of COPD remains an important goal given the huge personal and economic costs of the condition. Machine learning approaches, which can be tailored to an individual’s baseline profile and can learn from experience of the individual patient, show promise in achieving this goal. There is a need for larger datasets with which to develop more accurate algorithms; however, the lack of an effect of telehealth in COPD demonstrated in trials has effectively discouraged large implementations of the technology. One solution (if governance regulations can be overcome) is to amalgamate existing international datasets. Another may be to explore the ability of algorithms to predict moderate (nonhospitalized) exacerbations with all the challenges highlighted above. Additionally, the potential of machine learning to elucidate optimal interventions should be explored.

## Appendix

Multimedia Appendix 1 Supplementary data.

Multimedia Appendix 2 Receiver operating characteristic (ROC) of the multitask neural net (MTNN) and the symptom-counting exacerbation score (after [2]) for prediction of 24-hour admissions using the imputed data scenario. The areas under the mean aggregate ROC curves over test data are ~0.74 and ~0.52 respectively.

## Abbreviations

AUC: area under the receiver operating characteristic curve
COPD: chronic obstructive pulmonary disease
FEV _1_: forced expiratory volume in the first second of expiration
NHS: National Health Service
UK: United Kingdom
