# Supplementary material for: Improving Prediction of Risk of Hospital Admission in Chronic Obstructive Pulmonary Disease: Application of Machine Learning to Telemonitoring Data
Source: J Med Internet Res. 2018 Sep 21;20(9):e263. doi: 10.2196/jmir.9227 (PMC6231768; doi:10.2196/jmir.9227)
Supplement: Multimedia Appendix 1 [file jmir_v20i9e263_app1.pdf]

***Supplement***

*Identifying individuals at high risk of emergency admissions*

In the imputation scenario, at 80% sensitivity our algorithm resulted in the false positive rate of approximately 40%, which is nearly twice as good as that of the score counting methods. However, due to the imbalanced labels (the number of hospital admissions ≤0.1% of the sample size), the number of false positive detections may still be large, with the positive predictive value and the negative predictive value close to 0 and 1 for most calibrations. As an alternative initial evaluation of our machine learning algorithm for its possible use in anticipatory care, we computed risk ratios over the unseen test episodes, where we used the outputs of our algorithm to define the risk exposures, and the frequency of 24-hour admissions to define the outcomes. We found that, when the best machine learning models were tested on unseen data, 50% of all admissions occurred in the top 20% of the highest-risk patients as estimated by our algorithm, and 84% of all admissions occurred in the top 50% of the highest-risk patients. Patients classified as top 20% risk were 7.6 times as likely to have an admission than patients classified as the bottom 20% risk, and patients classified as above the median risk were 5.4 times as likely to have a COPD admission as patients classified as below the median risk. This suggests that, in a resource constrained environment, a triage strategy could be based on using the output of our method as an additional filtering criterion for prescribing preventive interventions.

*Cross-validation vs testing in multiple cohorts*

In this work, we evaluated the performance in accordance to the area under the ROC curve by aggregating the risk scores across the test folds and by averaging across multiple repetitions of cross-validation. This aggregated AUC criterion does not assume a perfect calibration of the model to the test data, and typically results in a lower estimate than averaging the areas under the ROC curves across the folds. (For instance, our estimates of the test AUCs increased by several points by changing the details of how the AUC is evaluated [1]. We have intentionally decided to report the more conservative estimates to account for the fact that the models may need to be tested in disparate populations with the varying frequencies of hospital admissions and outside of the controlled trial environment, where we may not be able to ensure a perfect calibration of the algorithm for any new replication cohort. Future work will account for the differences between the training and test scenarios by testing in multiple populations as suggested in the main text, which will help to circumvent the details of the evaluations using cross-validation.

*ROC curve for predictions of hospital admissions*

Multimedia Appendix 2 shows the mean ROC curves for the augmented test set of patient-episodes for independent patients for the prediction of 24-hour admissions computed for the trained machine learning model (based on a multi-task neural network) and the exacerbation-based symptom-counting score (based on Rodriguez-Roisin et al [2], definition 2, Figure 1 in the main paper). To avoid cluttering the plot, the 95% confidence intervals computed by resampling are shown for the machine learning model only (for the discussion of methodological limitations of error bars for cross-validation, see Bengio et al and Arlot et al [3,4] and the main paper). The machine learning algorithm achieves the true positive rate (sensitivity) of 0.80 at the false positive rate of

~0.40 (specificity 0.60), whereas the symptom-counting algorithm reaches this sensitivity at the false positive rate of ~0.80 (specificity 0.20).

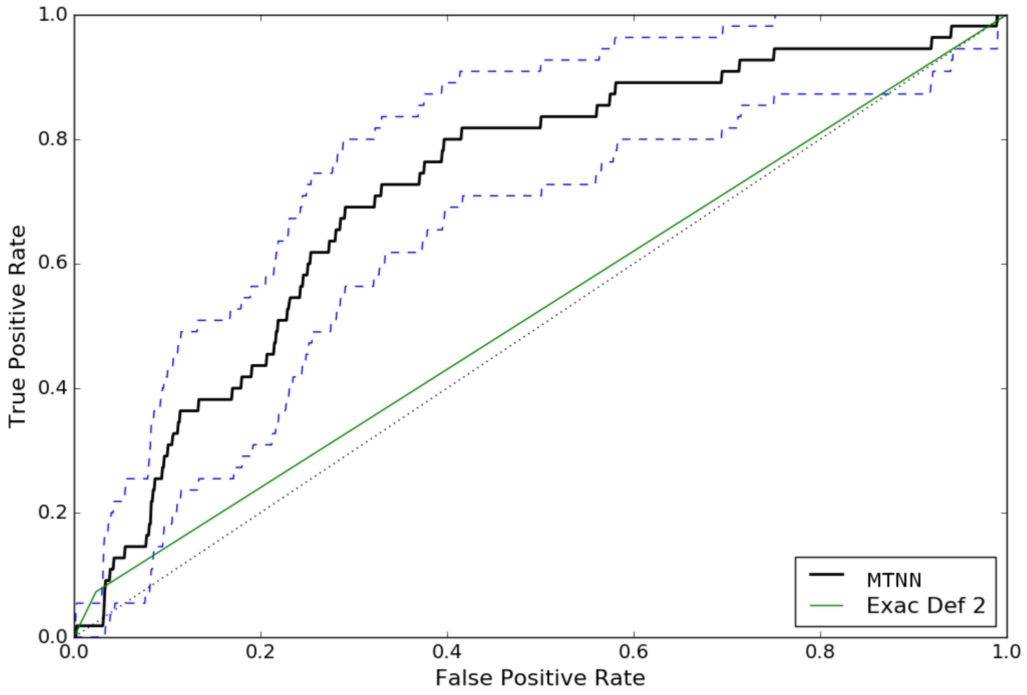


Multimedia Appendix 2 Receiver Operating Characteristic of the multi-task neural net (MTNN) and the symptom-counting exacerbation score (after (2)) for prediction of 24-hour admissions using the imputed data scenario. The areas under the mean aggregate ROC curves over test data are ~0.74 and ~0.52 respectively.

*Information extraction*

The telemonitoring database of was held by the NHS and linked in the Lothian safe haven with trial data held by the research team. Multiple information extraction packages could potentially be used to perform the linkage [5]; we have used standard spreadsheet software to manually link patient IDs to admission records (see Pinnock et al [6] for more details). Datasets were de-identified before analysis. The exported anonymized data was processed in *python v3*.

*Feature extraction*

We extracted 153 features from baseline characteristics and time series of self-reported symptoms, physiological measurements, and medications, known to be useful for monitoring COPD patients [6]. We note that it is not uncommon for machine learning methods to use hundreds or even thousands of features for predicting clinical events (e.g. [7,8]); in our case, we were limited by the small number of COPD-related admissions preceded by the periods where self-reported measurements were present ($N_+$=55 in the imputation scenario). The features included:

- the baseline static measurements (weight, height, age, FVC, FEV1, current smoking status, medication history, presence of co-morbidities, questionnaires: EuroQoL, HADS, MARS) assumed to be fixed during training;
- lagged self-reported symptoms (breathlessness, sputum colour, sputum amount, cold, wheeze, sore throat, cough, fever), physiology (heart rate and oxygen saturation), and medications (anti-biotic, steroid, reliever use) in the 5 days preceding the predicted events;
- trend, oscillator, and distribution features extracted from the time series by using computational methods [7,9] for the raw measurements and their differences over the 15-day window preceding the predicted events. The choice of 15-day windows (14 previous days and the day immediately preceding the admission or steroid onset) was motivated by the work of Seemungal et al. [10], who had demonstrated the presence of apparently consistent nonlinear trends in the time course of symptoms of increased dyspnoea, cough, and congestion over 14 days before onset of exacerbations on the population level. The choice of the distribution (histogram) features was motivated by the possible skewness and heavy tails of the marginal distributions of the continuous features.

In the imputation scenario, for the lagged variables we have additionally used missingness indicators to highlight whether the variables were reported or missing. The resulting variables were combined to learn additional features in the hidden layers (neural nets), used for computing feature-space similarity functions (non-parametric methods), or combined with feature selection by filtering to set priors on hyper-parameters during training for the adaptive regression methods (see below). When the output variables were used directly or indirectly to select or extract the features during training, we ensured that the procedure was nested within the training folds, so that the data used for the evaluations remained unseen.

*Feature selection and preprocessing*

We have standardized the continuous covariates to have zero mean and unit variance computed over the training folds only. We used one-hot encoding for categorical covariates. For all the models except the ensembles developed for the imbalanced tasks, we up-sampled the rare classes using Gaussian noise for the standardized continuous variables, and perturbation noise for the categorical variables.

For the computationally expensive and data-demanding neural nets, we used unsupervised feature selection by discarding all the variables highly correlated with other variables from the active set ($\rho^2 > 0.95$), starting from a random initialization. We did this in each training fold. A neural net refitted to the entire dataset following the model selection had 135 variables.

For the adaptive regularized models, we used feature selection by univariate filtering. For all variables *i* marginally significantly associated with the outcome according to the likelihood-ratio test, we set the regularization penalty to $\alpha_i = \alpha/|\beta_i|$, where $\beta_i$ was the variable's marginal effect size. For all the variables that were filtered out, we set the penalty to $\alpha_i = \alpha/\min_i |\beta_i|$. We then fitted an elastic net using all the variables *i*, with the corresponding penalties set to $\alpha_i$. We learned $\alpha$ by the line search, with evaluations over the inner folds of the nested cross-validation procedure.

We have not used feature selection for the considered ensembles and non-parametric methods, as neither the speed nor convergence of the algorithms were sensitive to possible collinearities in the features. These models used 153 features.

*Exemplar feature distributions*

Multimedia Appendix 2 visualizes the marginal distribution of oxygen saturation and heart rate across all the patient episodes in the training data, under the complete data setting. Table 1 summarizes the marginal distributions of the self-reported symptoms and medications under the complete data setting. We note that in this complete data setting we excluded all the episodes where at least one variable was missing. We refer the readers to Pinnock et al. [6], Tables 1-3 for other related summaries, including the patients' characteristics and questionnaires.

Multimedia Appendix 3. Marginal distributions of the heart rate on the left and oxygen saturation on the right computed for all the patient-episodes. The density plot is computed by kernel density estimation and is used in the computation of the histogram features

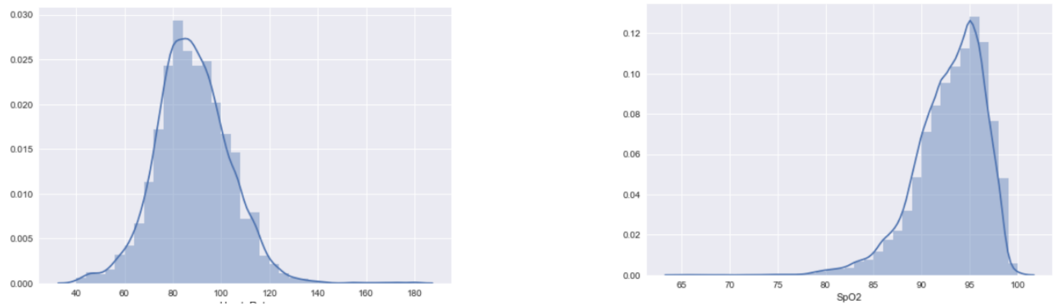


Table 1 (supplement) Marginal distributions of the self-reported symptoms and medication information. Note that some features are better balanced than the others (for example, breathlessness is a common symptom in this population, whereas fever is not).

| Self-reported symptoms | | | | |
|---|---|---|---|---|
| | nPos | nNeg | %Pos | %Neg |
| Sputum Colour | 2857 | 6911 | 29.249 | 70.751 |
| Sputum Amount | 776 | 8992 | 7.944 | 92.056 |
| Cold | 2456 | 7312 | 25.143 | 74.857 |
| Wheeze | 2333 | 7435 | 23.884 | 76.116 |
| Sore Throat | 620 | 9148 | 6.347 | 93.653 |
| Cough | 2852 | 6916 | 29.197 | 70.803 |
| Fever | 80 | 9688 | 0.819 | 99.181 |
| **Self-reported medications** | | | | |
| Reliever | 2876 | 6892 | 29.443 | 70.557 |
| Steroids | 238 | 9530 | 2.437 | 97.563 |
| Antibiotics | 1279 | 8489 | 13.094 | 86.906 |

*Predictions of multiple models*

Table 2 summarizes the augmented test AUC of multiple machine learning models for the prediction of 24-hour COPD admissions on independent test samples in the imputation setting. All the models

significantly outperformed the best of the symptom-counting scores with the test AUC of 0.524 [0.486, 0.544]. We note that we have only considered the settings that are instrumental for addressing the class imbalances, and we have applied structural and parametric regularization to avoid overfitting. Under these assumptions, the considered models performed approximately similarly; we therefore hypothesize that the observed improvements over the symptom-counting heuristics are achievable irrespective of the modelling assumptions (assuming the generalization is addressed carefully). There is no evidence of the substantial difference in the performance of the models, which is not surprising given the small number of predicted events. A single model that was marginally better than its competitors was the multi-task neural net. (In our setting, the target task corresponded to admissions on day *t+1,* the auxiliary tasks corresponding to admissions on days *t+2, …, t+7*, and features and hyper-parameters were shared across the tasks).

We used *python v3* and *keras* [11] (with the *TensorFlow* [12] back-end), *scikit-learn* [13], and *glmnet* [14] for fitting the models. To predict our secondary endpoint, steroid onset, we fitted the non-parametric classifier (it was the close second in performance for predicting our primary outcome, hospital admissions, but was more computationally effective).

Table 2 (sup) Prediction of 24-hour COPD-related hospital admissions using multiple models, for the imputation setting. All the models significantly outperform the exacerbation scores (the best result for the imputation setting is 0.524 [0.486, 0.544]).

| **Prediction of 24-hour admissions using machine learning models, *imputed* data** | | | | |
|---|---|---|---|---|
| **Description** | **Practical (Y/N)** | **AUC [Empirical 95% CIs]** | **Events, $N_+$** | **Samples, $N$** |
| Multi-task neural net | Yes | 0.740 [0.673, 0.803] | 55 | 57,150 |
| Non-parametric ($L_{1,2}$ SVM) | Yes | 0.738 [0.675, 0.802] | 55 | 57,150 |
| Ensemble (classification forest) | Yes | 0.732 [0.602, 0.794] | 55 | 57,150 |
| Adaptive regularized classifier | Yes | 0.721 [0.663, 0.779] | 55 | 57,150 |

*Predicting peaks in symptom scores with Healthy Outlook®*

Although the Healthy Outlook®[18] algorithm and the weather variables described earlier did not improve the quality of predictions of hospital admissions in our dataset, the weather variables may potentially be more promising for predicting variations in COPD symptoms at the population level. For each patient, we defined the symptom score to be the number of COPD symptoms present on that day. We defined the baseline-adjusted symptom score (BASS) to be the difference between the patient's symptom score on that day and the patient's mean symptom score for the first *m* days at and immediately after baseline. We assumed that fluctuations in BASS accounted for the variations from the "normal" level of symptoms near baseline; additionally, some adjustment of this kind was needed to combine data from patients with varying levels of symptoms. Our predictor variables were the lagged HO scores and the weather variables computed over the last two weeks. Our outcome was the two-week moving average of the future BASS scores averaged across the population. We used the nested cross-validation with 10 inner and 10 outer folds to investigate whether an elastic net using the weather variables and the Healthy Outlook® scores improved the predictions over a simple heuristic approximating the next population-level two-week average BASS outcome to be equal to such last observed outcome (the naïve lagged predictor). Particular care was

taken in ensuring that there was no overlap between the labels in the inner- and outer-test/validation folds and the training folds for the longer-term time series predictions. To adjust for the average baseline symptom scores, we used *m*=20. This value was chosen from a small independent candidate set after evaluating the best coefficient of determination for next day BASS predicted from temperature. We found that over some contiguous time periods predominantly during autumn and winter, prediction of the two-week population-averaged BASS using the Healthy Outlook® variables outperformed the prediction of the simple delayed BASS, with the Spearman correlation between the true and the predicted outcome over the test data folds increasing from 0.44-0.55 (the lagged heuristic) to 0.66-0.75 (Healthy Outlook®), and with the Kendall rank correlation increasing from 0.27-0.38 to 0.44-0.52. However, the advantage of the HO over the naïve predictions seemed to have disappeared over the periods including warmer months, and the results were sensitive to the adjustment for baseline *m*. This suggests that despite the observation that the HO score and the weather variables did not improve individual predictions in this study, there may be some potential use in predicting spikes in COPD symptoms on the population level over the longer future periods, which requires further investigations.

*Bibliography*


1. Forman G, Scholz M. Apples-to-apples in cross-validation studies: pitfalls in classifier performance measurement. ACM SIGKDD Explor Newsl. 2010;12(1):49–57. Available from http://www.kdd.org/exploration_files/v12-1-p49-forman-sigkdd.pdf (Archived at WebCite http://www.webcitation.org/6ydtyw7Au

2. Rodriguez-Roisin R. Toward a consensus definition for COPD exacerbations. Chest. 2000 May;117(5):398S–401S. PMID:10843984

3. Bengio Y, Grandvalet Y. No unbiased estimator of the variance of k-fold cross-validation. J Mach Learn Res. 2004;5(Sep):1089–1105. Available from www.jmlr.org/papers/volume5/grandvalet04a/grandvalet04a.pdf (archived at WebCite http://www.webcitation.org/6yduJZJbZ)

4. Arlot S, Celisse A. A survey of cross-validation procedures for model selection. Stat Surv. 2010;4:40–79. Available from https://www.di.ens.fr/willow/pdfs/2010_Arlot_Celisse_SS.pdf (archived at WebCite http://www.webcitation.org/6yduTIy8g)

5. Wang Y, Wang L, Rastegar-Mojarad M, Moon S, Shen F, Afzal N, et al. Clinical information extraction applications: A literature review. J Biomed Inform. 2018 Jan;77:34–49. PMID: 29162496

6. Pinnock H, Hanley J, McCloughan L, Todd A, Krishan A, Lewis S, et al. Effectiveness of telemonitoring integrated into existing clinical services on hospital admission for exacerbation of chronic obstructive pulmonary disease: researcher blind, multicentre, randomised controlled trial. BMJ 2013 Oct; 347:f6070 PMID:24136634

7. Che Z, Purushotham S, Cho K, Sontag D, Liu Y. Recurrent neural networks for multivariate time series with missing values. ArXiv Prepr ArXiv160601865. 2016; Available from https://arxiv.org/pdf/1606.01865

8. Oh J, Makar M, Fusco C, McCaffrey R, Rao K, Ryan EE, et al. A generalizable, data-driven approach to predict daily risk of Clostridium Difficileinfection at two large academic health centers. Infect Control Amp Hosp Epidemiol. 2018 Apr;39(4):425–33. PMID:29576042

9. Caruana R, Lou Y, Gehrke J, Koch P, Elhadad N. "Intelligible models for healthcare: Predicting pneumonia risk and hospital 30-day readmission". *Proceedings of the 21st ACM SIGKDD International Conference on Knowledge Discovery and Data Mining*. pp 1721-30 ACM 2015 ISBN: 978-1-4503-3664-2 ( Archived by WebCite at **http://www.webcitation.org/6ycTdjTJA**)

10. Seemungal TAR, Donaldson GC, Bhowmik A, Jeffries DJ, Wedzicha JA. Time course and recovery of exacerbations in patients with chronic obstructive pulmonary disease. Am J Respir Crit Care Med 2000a May; 161:1608–13. PMID:10806163

11. Chollet F, Keras [Software]. 2015. Available from: https://keras.io/

12. Abadi M, TensorFlow: Large-Scale Machine Learning on Heterogeneous Systems [Software]. 2015. Available from: https://www.tensorflow.org/

13. Pedregosa F, Varoquaux G, Gramfort A, Michel V, Thirion B, Grisel O, et al. Scikit-learn: Machine learning in Python. J Mach Learn Res. 2011;12(Oct):2825–2830. Available from http://www.jmlr.org/papers/volume12/pedregosa11a/pedregosa11a.pdf (archived by WebCite at http://www.webcitation.org/6ydvUS9FT)

14. Friedman J, Hastie T, Tibshirani R. Regularization Paths for Generalized Linear Models via Coordinate Descent. J Stat Softw. 2010;33(1):1–22. PMID:20808728
