# Supplementary figures and images for: Improving Prediction of Risk of Hospital Admission in Chronic Obstructive Pulmonary Disease: Application of Machine Learning to Telemonitoring Data
Source: J Med Internet Res. 2018 Sep 21;20(9):e263. doi: 10.2196/jmir.9227 (PMC6231768; doi:10.2196/jmir.9227)

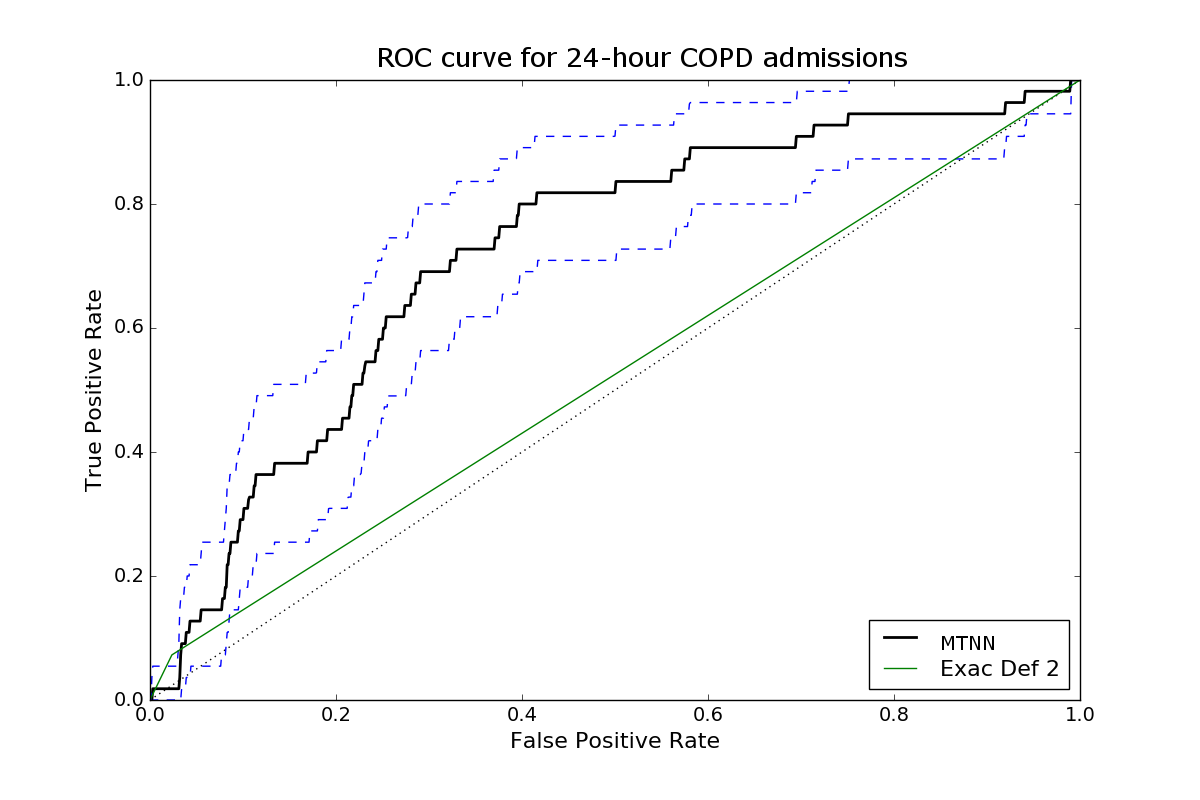

Supplement: Multimedia Appendix 2 [file jmir_v20i9e263_app2.png]
